# Supplementary material for: A set of multi-entry identification keys to African frugivorous flies (Diptera, Tephritidae)
Source: Zookeys. 2014 Jul 24;(428):97–108. doi: 10.3897/zookeys.428.7366 (PMC4143993; doi:10.3897/zookeys.428.7366)
Supplement: Supplementary material 6 — Key to Ceratitis [file zookeys-428-097-s006.zip › SF6_ZooKeys_key to Ceratitis/key/SF6_ZooKeys_key to Ceratitis/Media/Html/Ceratitis perseus.htm]

***Ceratitis*** *(**Ceratalaspis**)
**perseus*** **De Meyer & Copeland**

*Ceratitis (Ceratalaspis) perseus* De Meyer
& Copeland, 2005 : 1291.

Body
length. 5.60 (4.80-6.20) mm; wing length. 5.35 (4.55-5.70) mm.

Male

Head.
Antenna yellow to yellow-orange. First flagellomere 3 times as long as pedicel.
Arista with medium long rays, longest dorsal rays about 3 times as long as
basal width of arista; ventral rays shorter than dorsal rays basally. Frons
yellow to pale yellow; with short scattered black and yellow setulae. Face white.
Genal seta and setulae black, well developed.

Thorax.
Postpronotal lobe pale yellowish white; with black spot. Scutal pattern, ground
colour yellow to dark orange, with distinct black markings, including median
sutural spot, prescutellar markings white, separated by pale area; with golden
microtrichosity and yellow pilosity. Scapular setae black. Scutellum white to
yellow, basally with black spots, apically with three separate black spots,
extending to basal third. Subscutellum black. Anepisternum yellow, upper half
white, one anepisternal seta, setulae white to yellow.

Legs.
Yellow; setation yellow to orange. Fore femur with ventral setae mainly black;
posteriorly with dense yellow to silvery pilosity. Mid leg, femur ventrally
with few conspicuously longer yellow setulae along basal third; tibia with
black feathering at apical outer half and inner two-fifth, area of tibia with
feathering coloured black.

Wing.
Bands mainly yellowish with some brown patches. Anterior apical and discal
bands largely but incompletely separated, narrowly joined; subapical band free;
posterior apical band absent; crossvein R-M at, or just distal to, middle of
discal medial cell.

Abdomen.
Yellow to orange; pilosity mixed yellow and black. Tergites 2 and 4 with pale
grey band occupying posterior third of tergite. Tergite 5 with longer black
setulae along posterior margin.

Female

As
male except for the following characters. Frons only with short black setulae.
Fore femur with less dense pilosity posteriorly; mid tibia without black
feathering, completely yellow. Oviscape shorter than preabdomen. Aculeus 8
times longer than wide, tip with median step and lateral margin slightly
sinuate.

(Description
after De Meyer & Copeland, 2005)
